# Mechanistic Insights into Electronic Current Flow through Quinone Devices

**DOI:** 10.3390/nano13243085

**Published:** 2023-12-05

**Authors:** Lawrence Conrad, Isaac Alcón, Jean Christophe Tremblay, Beate Paulus

**Affiliations:** 1 Institut für Chemie und Biochemie, Freie Universität Berlin, 14195 Berlin, Germany; 2 Catalan Institute of Nanoscience and Nanotechnology (ICN2), Consejo Superior de Investigaciones Científicas (CSIC) and Barcelona Institute of Science and Technology (BIST), Campus Universitat Autònoma de Barcelona (UAB), Bellaterra, 08193 Barcelona, Spain; isaac.alcon@icn2.cat; 3 Laboratoire de Physique et Chimie Théoriques, Centre National de la Recherche Scientifique (CNRS)-Université de Lorraine, 1 Bd Arago, 57070 Metz, France; jean-christophe.tremblay@univ-lorraine.fr

**Keywords:** non-equilibrium Green’s function, Landauer formula, quinones, local currents, graphene nanoribbons, nanoelectronics

## Abstract

Molecular switches based on functionalized graphene nanoribbons (GNRs) are of great interest in the development of nanoelectronics. In experiment, it was found that a significant difference in the conductance of an anthraquinone derivative can be achieved by altering the pH value of the environment. Building on this, in this work we investigate the underlying mechanism behind this effect and propose a general design principle for a pH based GNR-based switch. The electronic structure of the investigated systems is calculated using density functional theory and the transport properties at the quasi-stationary limit are described using nonequilibrium Green’s function and the Landauer formalism. This approach enables the examination of the local and the global transport through the system. The electrons are shown to flow along the edges of the GNRs. The central carbonyl groups allow for tunable transport through control of the oxidation state via the pH environment. Finally, we also test different types of GNRs (zigzag vs. armchair) to determine which platform provides the best transport switchability.

## 1. Introduction

Since its discovery in 2004, [1] graphene has sparked immense interest for its interesting electronic properties [2,3,4,5]. Due to its extraordinary conducting properties, there has been a large interest in its applications and the nanomaterials that can be derived from it. Narrow strips of graphene can be produced by cutting graphene or carbon nanotubes or through more sophisticated bottom-up synthetic methods to produce graphene nanoribbons [6,7,8,9]. Depending on the edge structure these can be divided into metallic zigzag graphene nanoribbons (ZGNRs) and mostly semi-metallic to metallic armchair graphene nanoribbons (AGNRs) [10,11,12]. This produces quasi-1D materials that can produce versatile electronic properties through functionalization [13,14]. Uses can include transport devices, but also biosensing materials [15,16]. The production of larger ZGNRs has been a challenge for many years due to its inherent spin polarization, [17] although much progress has been made in recent years [18,19,20,21,22]. AGNRs are considerably more stable and therefore easier to produce experimentally [8,23,24].

The use of functionalization to modulate conductance according to the pH environment and therefore act as a pH-sensitive switch has been proposed in various works [25,26,27]. In 2012, Darwish et al. discovered a connection between the conductance of an anthraquinone derivative and the acidity of the environment [28]. This was found to be due to the influence of the pH value on the quinone-hydroquinone equilibrium. At pH 3 the reduction of the quinone was found to be cathodically shifted over the reduction at pH 8. The hydroquinone was found to be more conducting and therefore, over a potential window at lower pH values, the constructed junction is more conducting than at higher pH values. A similar structure designed for attachment to nanowires was published soon afterwards [29]. In previous work it had already been proposed that anthraquinone moieties would exhibit considerably less conductance than their reduced forms due to a destructive quantum interference effect [30]. Since then, larger network of molecular junctions based on this structure have been investigated [31,32]. Quinones have also been used to tune the conductivity of Mo_2_ and WS_2_ and the optoelectronic properties of carbon dots [33,34]. Additionally, similar pH based switches have been proposed for electrochemically tunable nanoporous graphene [35]. The pH dependent shift in the equilibrium between quinones and hydroquinones is thus already established as a strong design principle for moleular switches.

In this work, we examine the electronic and transport properties of both an example for a ZGNR and an AGNR, functionalized with a quinone group or two carbonyl groups respectively. For simplicity and consistency in the following text, the latter shall be referred to as 5-AGNR systems in quinone or hydroquinone form. The electronic structure is investigated through methods of density functional theory (DFT) and the resulting Hamiltonian matrices are extracted. These are used in the calculation of the transport properties through nonequilibrium Green’s function (NEGF) and the Landauer formalism. A procedure first proposed by Evers and co-workers enables the representation of the local current flow [36,37,38,39,40,41]. These local currents provide invaluable mechanistic information about the charge transport mechanism under non-equilibrium conditions and have allowed establishing structure-function relationships in other graphene-based systems [16,42,43,44]. An effect that is related but not investigated here is the pH dependent shift of the drain-source current gate potential curve that is observed in ZGNRs [45].

## 2. Methods

### 2.1. Models

To investigate the potential of quinone-derivatives as pH-selective switches, three separate devices were studied: one based on a quinone functional unit integrated in a 2-ZGNR, and two with two carbonyl groups functionalizing a 5-AGNR, either in *cis* and *trans* configuration. At low pH, equilibrium will be skewed towards those anthraquinone derivatives, where both carbonyl groups will accept a hydrogen atom [46]. This in turn influences the potential required to change the oxidation state. The balance between these species will depend on the chemical environment, which can be precisely controlled experimentally. In this work, we thus only investigate the quinone and anthraquinone forms as limiting cases for the transport mechanism in low (below 5) and high pH (above 8) environments. While these investigations are not quantitative, they allow to extract mechanistic information about electrons flowing through the device under quasi-static transport conditions.

**Figure 1:**
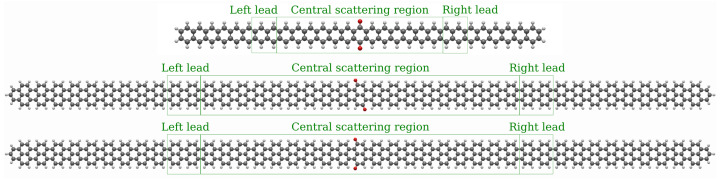
Structure of investigated systems in oxidized form. From top to bottom: the 2-ZGNR with a central quinone unit, 5-AGNR with carbonyl groups in *trans* and in *cis* configurations. The parts that were selected for the central scattering region, as well as the left and right leads are marked with green boxes. Carbon atoms are drawn in black, hydrogen atoms in white, and oxygen atoms in red.

For the calculation of the electronic and transport properties these were placed between units of the corresponding 2-ZGNR and 5-AGNR to form extended polyaromatic hydrocarbon molecules which act as leads. These 5-AGNR leads were selected, because AGNR leads show a relatively small band gap according to the 3p+2 rule. Ref. [12] For the transport simulations, the semi-infinite leads were constructed from the units halfway between the central scattering region and the ending groups, as shown in Figure 1. The size of the buffer zone between the leads and the scattering region was increased to the point, where the eigen energies for the two units comprising the leads converged below an energy threshold of $10^{-2}$ eV with a maximum of two outliers below $3\times 10^{-2}$ eV being tolerated. More units are required to converge the AGNR than the ZGNR. Hence, the investigated systems are of different lengths with 6 units on either side between the scattering region and the leads for the ZGNR, and 9 units for the AGNR systems. For further details on the model construction, see the Supporting Information. According to this partitioning of the system, the Hamiltonian matrix is also split into diagonal blocks $H_{C}$, $H_{L}$, and $H_{R}$ for the central scattering region, left lead, and right lead, respectively. The coupling between the local Hamiltonians is described by off-diagonal blocks $H_{LL}$, $H_{LC}$, $H_{CR}$ and $H_{RR}$. The leads blocks and their coupling elements are repeated to form semi-infinite leads on both sides.

(1) $$H=\left(\begin{array}{ccccccc} \ddots & H_{LL} & 0 & 0 & 0 & 0 & 0\\ H_{LL} & H_{L} & H_{LL} & 0 & 0 & 0 & 0\\ 0 & H_{LL} & H_{L} & H_{LC} & 0 & 0 & 0\\ 0 & 0 & H_{LC} & H_{C} & H_{CR} & 0 & 0\\ 0 & 0 & 0 & H_{CR} & H_{R} & H_{RR} & 0\\ 0 & 0 & 0 & 0 & H_{RR} & H_{R} & H_{RR}\\ 0 & 0 & 0 & 0 & 0 & H_{RR} & \ddots \end{array}\right)$$

The overlap matrix *S* is treated in a similar fashion. From the diagonal blocks of the Hamiltonian and overlap matrices, it is possible to determine the local energy spectrum of a chosen unit *i* by solving the secular equation in matrix form $H_{i}C_{i}=S_{i}C_{i}E_{i}$, where $C_{i}$ is the matrix of coefficients of the eigenvectors associated with the matrix of eigenenergies $E_{i}$.

A bias $V_{bias}=\left(\mu_{L}-\mu_{R}\right)/2$ is applied to induce the current, where $\mu_{L/R}$ are the chemical potentials of the leads. At positive bias, this results in an increase of the local state populations in the left lead and a decrease on the right lead populations. The probability of electrons travelling from the left lead to the right is expressed by the transmission function, which can be derived from the advanced and retarded Green functions

(2) $$T\left(E\right)=Tr[G^{r}\left(E\right)\Gamma_{L}\left(E\right)G^{a}\left(E\right)\Gamma_{R}\left(E\right)]$$

Here, $\Gamma_{L/R}$ are the spectral broadening matrices $\Gamma_{L/R}=i\left(\Sigma_{L/R}-\Sigma_{L/R}^{†}\right)$. $G^{r}$ is the retarded Green’s function, given as $G^{r}\left(z\right)={\left(zS_{C}-H_{C}-\Sigma_{L}\left(z\right)-\Sigma_{R}\left(z\right)\right)}^{-1}$ with $z=E+i0^{+}$. The advanced Green’s function $G^{r}$ is the same function with $z=E-i0^{+}$. $H_{C}$ and $S_{C}$ here are the Hamiltonian and overlap matrices for the central scattering region and $\Sigma_{L/R}$ are the self-energy terms of the leads.

The global current is calculated using the Landauer formula

(3) $$I_{e}=-\frac{2e}{h}\int_{-\infty }^{+\infty }T\left(E\right)\left(f_{L}\left(E\right)-f_{R}\left(E\right)\right)dE$$

with *e* as the elementary charge, *h* as the Planck constant and $f_{L/R}$ as the Fermi distribution for the left and right leads, respectively. The factor two accounts for the degeneracy of the two different spin channels. The lesser Green’s function takes the form

(4) $$G^{<}\left(E\right)=iG^{r}\left(E\right)\left(f_{L}\left(E\right)\Gamma_{L}\left(E\right)+f_{R}\left(E\right)\Gamma_{R}\left(E\right)\right)G^{a}\left(E\right)$$

From Equation (4), it was shown how local currents can be computed in real space from the current density operator as [36]

(5) $$j\left(r,E\right)=\frac{e}{2\pi }\frac{\hbar }{m}\sum_{A,B}^{}\sum_{\mu_{A},\mu_{B}}^{}\psi_{\mu_{A}}\left(r\right)G^{as}\nabla \psi_{\nu_{B}}\left(r\right)$$

where *ℏ* is the reduced Planck constant, *m* the resting mass of an electron, and $\psi_{\mu_{A}/\nu_{B}}\left(r\right)$ the atomic orbital basis functions projected on a spatial grid. Since atomic orbitals are strongly localized around atoms, in previous work it was showed that storing and using only values larger than a chosen threshold in Compressed Row Storage format can lead to important numerical savings [16]. We also noticed that the asymmetric Green’s function $G^{as}=\left(G^{<}-{\left(G^{<}\right)}^{T}\right)$ has limited spectral information, and its calculation can be made computationally more efficient by using the reduced singular value decomposition according to [16]

(6) $$G^{as}=USV^{T}$$

Here, only the ∼50 largest singular values (the diagonal elements of *S*) associated with singular vectors *U* and *V*, are kept. The total local current reported in this work can be obtained by integrating the current density over the energy range defined by the potential bias $V_{\mathrm{bias}}$ as

(7) $$J\left(r\right)=2\int_{E_{f}-V_{\mathrm{bias}}/2}^{E_{f}+V_{\mathrm{bias}}/2}j\left(r,E\right)dE$$

where $E_{f}$ is the Fermi energy and the factor 2 again comes from the degenerate spin channels. For further mechanistic insights the velocity fields are calculated from the local currents J(r) and the electronic density ρ(r) as

(8) v(r)=J(r)/ρ(r)

### 2.2. Computational Details

To represent the devices embedded between semi-infinite leads, molecular clusters were built out of repeated GNR cells. The center around the quinone structure and the hydrogen atoms on the edges were structurally optimized. Structural optimization of the hydrogens on the edges and of the central region were performed through the libxc implementation of the optPBE-vdW functional [47,48,49].

Systems based on AGNRs were investigated at the PBE/DZP level of theory, [50] using the GPAW package in LCAO mode [51,52]. Fermi smearing was used throughout together with a 7.0 Å vacuum in the transverse transport directions. This setup was found to be best suited to describe 5-AGNR systems and capture their small gap semiconductor character.

Calculations for the ZGNR systems were performed at the PBE0+D3/DZP [53,54,55,56] level of theory using the TURBOMOLE software package version 7.4 [57]. Inspired from findings in literature [12,58,59,60] and by tests conducted by the authors, it was concluded that this functional is preferable to PBE for these systems [61]. In the small gap AGNR 3p+2 systems, PBE0 was found however to open an artificial gap, and the PBE functional was preferred. Additionally, Fermi smearing was observed in the *cis* AGNR quinone system with occupancies of 1.82 and 0.18 electrons in the HOMO and LUMO respectively, which could be captured by GPAW. ZGNR systems of small widths can show spin polarization along the edges, which can be captured with unrestricted DFT methods [17,18]. For the systems studied in this work, restricted DFT calculations were found to yield the same energies and orbitals around the Fermi energy.

The electronic transport calculations were performed using the transport module from Atomic Simulation Environment (ASE), which is based on the NEGF formalism [62,63,64,65,66]. Orbitals and their derivatives were extracted and processed with the ORBKIT program package. Refs. [67,68,69] ORBKIT was also used to project the atomic orbitals and their derivatives onto a Cartesian grid to plot the local currents.

For the 2-ZGNR systems the Hamiltonian matrices are reconstructed from the molecular orbitals and overlap matrices obtained from the electronic structure calculations performed using TURBOMOLE. A detailed explanation of the extraction procedure is given in the Supporting Information. The Hamiltonian and overlap matrices for the 5-AGNR systems are extracted directly through ASE, which natively supports GPAW. The transport properties were investigated for a bias window between 0.0–1.5 V.

## 3. Results and Discussion

### 3.1. Devices Based on Zigzag Graphene Nanoribbons

**Figure 2:**
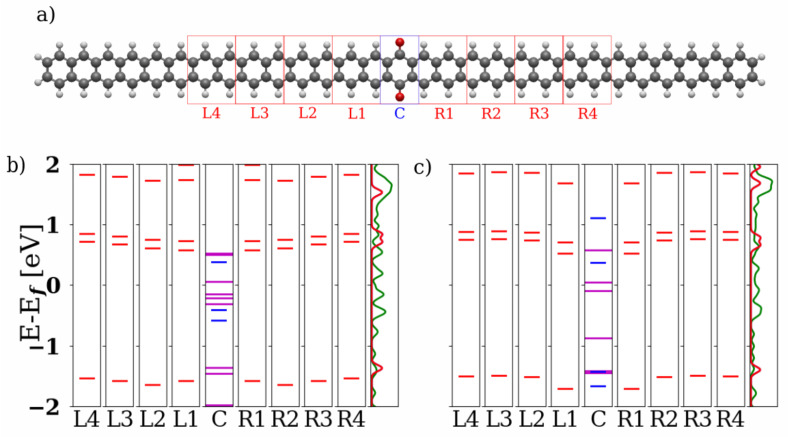
(**a**) The structure and partitioning of the 2-ZGNR quinone system is depicted above. The carbon atoms are drawn in black, the hydrogen atoms in white and the oxygen atoms in red. The parts that were selected for the central scattering region (C) and the left (L1–L4) and right (R1–R4) cells are marked with red boxes in the image. (**b**) Local energy spectrum of the quinone structure and (**c**) local energy spectrum of the hydroquinone structure. The local states are labeled with the corresponding sections. The rightmost sections show the projected density of states (PDOS) for the leads in red and the complete cluster in green.

In Figure 2, local energy spectra of the 2-ZGNR systems both in the quinone and hydroquinone forms are depicted. For this, the investigated clusters and the corresponding Hamiltonian matrix blocks were partitioned as shown in the top panel. The eigenenergies in the respective regions were calculated and aligned at the Fermi energy $E_{f}$. For the 2-ZGNR systems, we observe that the alignment of the local energies in the conduction band of the hydroquinone form (right panel) is very good and therefore produces no impediment for transport. Both forms however achieve strongly shifted levels and with them better alignment through hybridization of the central region with the units L1 and R1 to either side. For these the coupling elements of the Hamiltonian and overlap matrices between the different sections play a larger role. The resulting states are depicted as longer purple lines in the section C. This hybridization over the entire cluster leads to several states lying between the local HOMOs and LUMOs for the leads as shown in the PDOS. This should provide transport channels for both forms. For more details on the hybridization see Section S4 of the Supporting Information.

**Figure 3:**
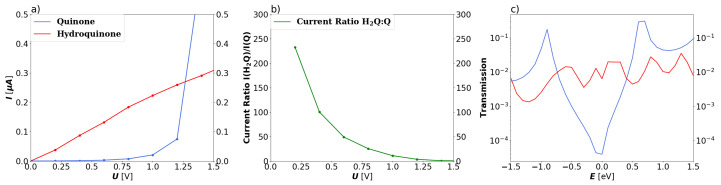
Global transport properties of the 2-ZGNR systems as a function of the applied bias voltage *U*. (**a**) Total current *I* calculated through the Landauer equation for the quinone (blue) and hydroquinone (red). (**b**) Ratio of total currents ($I_{H_{2}Q}/I_{Q}$) from hydroquinone to quinone systems. (**c**) Transmission function *T* over the investigated energy window of the quinone (blue) and hydroquinone (red) 5-AGNR systems.

In panel (a) of Figure 3, comparison of the total current of the 2-ZGNR systems as calculated by means of the Landauer equation is depicted. For the bias window between 0.0–1.2 V, the hydroquinone form exhibits a linear increase in conductance. In this window, the current ratio between the two forms, as shown in the right panel of Figure 3 (see also the current difference in the Supporting Information) indicate that the hydroquinone is clearly preferable for transport over the quinone form. The ratio remains higher than 10:1 for biases up to 1.0 V, at which point the current flow is considerably stronger than at 0.2 V, at which the ratio of the currents between the two forms is highest. At higher biases the quinone form becomes more advantageous for transport with a steep increase in current between 1.2 and 1.5 V. This correlates very well with the transmission functions shown in panel (c). The transmission funtions match very well with the ones calculated by Markussen et al. for their anthraquinone moiety between metallic leads. Ref. [30] For the hydroquinone form, it shows various shallow peaks and troughs always around $10^{-2}$. Meanwhile, the transmission function for the quinone form has a gap around the Fermi level, immediately rising steeply to either side and peaking above that of the hydroquinone form. This demonstrates that theory predicts that the destructive interference is also present in a functionalized 2-ZGNR.

The HOMO-LUMO gaps from the electronic structure calculations for the whole 2-ZGNR clusters are 0.573 and 0.448 eV for the quinone and hydroquinone, respectively. Note that the HOMO and LUMO energies of the delocalized systems are different than those in Figure 2, which reports local orbital energies on each unit. The marginal difference between the two systems cannot account for their distinctive behaviour. The local energy spectra shown in Figure 2 provide part of the answer. The perfect level alignment in the hydroquinone system would likely lead to delocalization of the frontier orbitals over the whole cluster (leads and scattering region), which could explain the observed transport behaviour reminiscent of metallic systems. The band alignment is not as favorable for the quinone.

**Figure 4:**
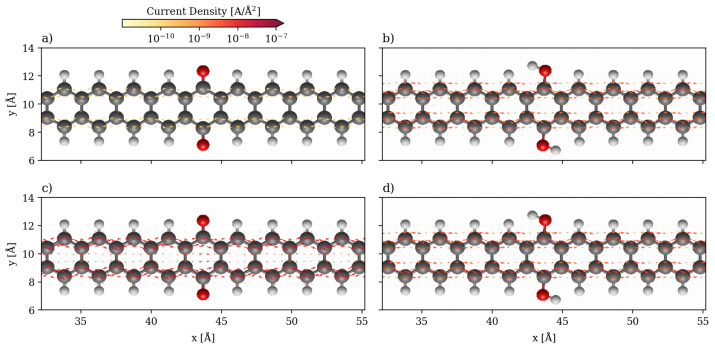
Quiver plot of the current densities in the 2-ZGNR systems. They are projected onto real space on a cartesian grid at 1 $a_{0}$ spacing in all directions. Only the central scattering region is displayed (same color code as in Figure 1). (**a**) Quinone system at 1.0 V. (**b**) Hydroquinone system at 1.0 V. (**c**) Quinone system at 1.5 V. (**d**) Hydroquinone system at 1.5 V.

The local currents for the 2-ZGNR systems are shown in Figure 4 at 1.0 V, at which the hydroquinone is more conducting, and 1.5 V, at which point the quinone is more conducting. At 1.0 V, the current flows only along the two edges independently and scattering from the quinone group greatly slows down the flow in comparison to the hydroquinone. The two carbonyl groups can be assumed to produce a potential barrier for the electrons travelling along the edges. Although the pathways the currents take are the same, there is a difference in the strength of current of about one order of magnitude, which corresponds to the ratio seen in the global currents. At 1.5 V, at which point the quinone is more conducting than the hydroquinone, the scattering is less impactful as it appears that non-negligible through-space transport occurs in the ring between the two carbonyls. Additionally, the destructive interference no longer plays a role. In contrast, the local currents are qualitatively the same for the hydroquinone both at 1.0 and 1.5 V, only more intense at the higher bias. They flow strictly along both edges as two independent channels.

**Figure 5:**
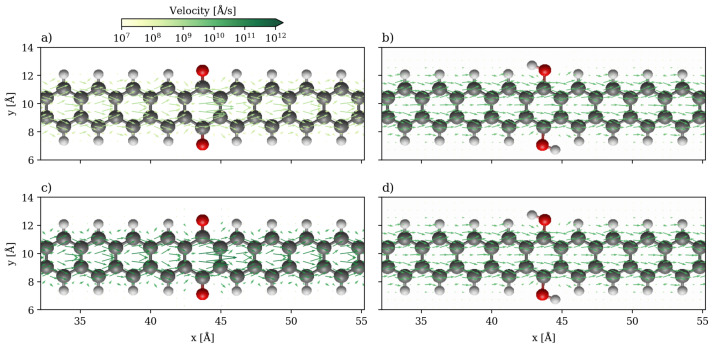
Quiver plot of the velocity fields in the 2-ZGNR systems. They are projected onto real space on a grid at 1 $a_{0}$ spacing. The central scattering regions are displayed with the same color-code as Figure 1. (**a**) Quinone system at 1.0 V. (**b**) Hydroquinone system at 1.0 V. (**c**) Quinone system at 1.5 V. (**d**) Hydroquinone system at 1.5 V.

In the velocity field plots, which are depicted in Figure 5, it can be seen that while the electrons in the hydroquinone system also travel along the edges at the fastest rate, the electrons in the quinone system travel both along the edges and through the empty space in the center.

This through-space transport mechanism is also observed in hydroquinone but to a lesser extent. On the other hand, the highest velocities are achieved in the center of the quinone ring at the point where the potential barrier produced by the quinone is crossed. This may be caused by a bottleneck effect as the carbonyl groups pose barriers to the transport along the carbon chain making this through-space transport the path of least resistance. At 1.5 V this pathway is also still the one that achieves the greatest velocities. The sharp change in the intensity of the currents in the quinone while the shapes remain the same may be a consequence of the quantum interference. At higher biases, the conductivity increases with the increase in the transmission function (see Figure 3), and the features of the quantum interference disappears from the local transport and velocity maps.

**Figure 6:**
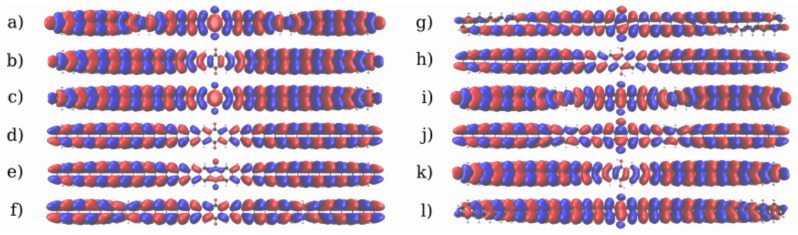
Frontier molecular orbitals of the 2-ZGNR systems. (**a**–**f**): The frontier orbitals of the quinone form from LUMO+2 to HOMO-2. (**g**–**l**): The frontier orbitals for the hydroquinone form from LUMO+2 to HOMO-2. The phases of the orbitals are depicted in red and blue.

To understand the origin of the barrier observed in the quinone, the frontier molecular orbitals of the two systems are shown in Figure 6. In both systems, the frontier orbitals are all mostly delocalized through the π orbitals. All orbitals of the hydroquinone are completely delocalized through the whole system, with significant amplitude in the central region. These orbitals are likely to favor ballistic transport, which explains the transport behaviour observed in the IV-curve. Interestingly, the HOMO of the quinone has a node in the center, which does not appear in the hydroquinone, and both the HOMO and HOMO-1 also only have small lobes in the center. That is the coupling among them is too small to favor delocalization of the orbitals through the whole device. While most of the reduced conductance can be attributed to the destructive interference, this could contribute to the appearance of a potential barrier and the reduced current flow at 1.0 V. For the quinone system, unhindered ballistic electron transport would necessitate the involvement of orbitals at higher and lower energies. These channels could become accessible at higher biases.

### 3.2. Devices Based on Armchair Graphene Nanoribbons

**Figure 7:**
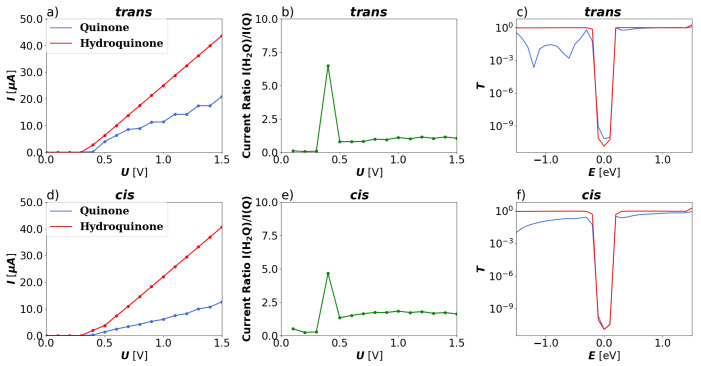
Global transport properties of the 5-AGNR systems as a function of the applied bias *U*. (**a**) Total current *I* calculated through the Landauer equation for the *trans* quinone (blue) and hydroquinone (red) 5-AGNR systems. (**b**) Ratio of total currents ($I_{H_{2}Q}/I_{Q}$) from hydroquinone to quinone systems of *trans* 5-AGNR systems. (**c**) Transmission function *T* over the investigated energy window of the *trans* quinone (blue) and hydroquinone (red) 5-AGNR systems. (**d**) Total current *I* for the *cis* quinone (blue) and hydroquinone (red) 5-AGNR systems. (**e**) Ratio of total currents ($I_{H_{2}Q}/I_{Q}$) from hydroquinone to quinone systems of *cis* 5-AGNR systems. (**f**) Transmission function *T* over the investigated energy window of the *cis* quinone (blue) and hydroquinone (red) 5-AGNR systems.

**Figure 8:**
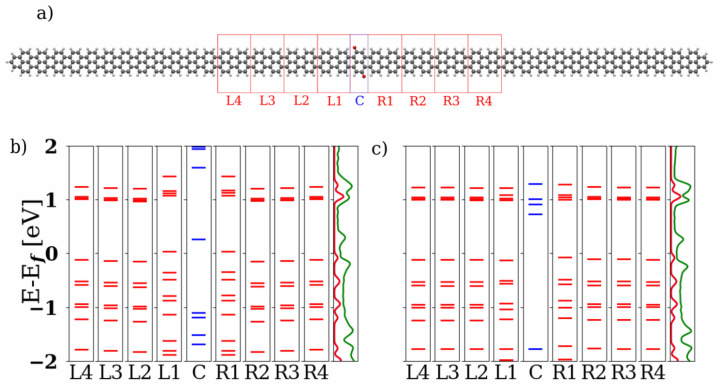
(**a**) Structure and partitioning of the *trans* 5-AGNR quinone system is depicted above. The carbon atoms are drawn in black, the hydrogen atoms in white and the oxygen atoms in red. The parts that were selected for the central scattering region (C) and the left (L1–L4) and right (R1–R4) cells are marked with red boxes in the image. Below: (**b**) The local energy spectrum of the quinone structure is shown and (**c**) the local energy spectrum of the hydroquinone structure. The local states are labeled with the corresponding sections. The rightmost sections show the PDOS for the leads in red and the complete cluster in green.

In contrast to the 2-ZGNR systems, the qualitative difference in conductance between the quinone and hydroquinone form remains the same at all investigated biases. For the *trans* configured 5-AGNR systems, throughout the bias window that is examined, the hydroquinones exhibit a higher total current than the quinones, as seen in Figure 7. The hydroquinone systems become conducting at a bias of 0.4 V and the quinone at 0.5 V. This creates a peak in the ratio between the currents at 0.4 V as seen in Figure 8. At higher biases the ratio fluctuates between 2–3. After crossing their respective thresholds both systems exhibit a linear increase in current. This is also reflected in the transmission functions shown in panels (c) and (f) of the same figure. In all cases there is a gap in the transmission function around the Fermi level with sharp increase in either direction. The transmission function of the quinone forms after the increase remain under those of the hydroquinone forms and show considerably lower levels at negative energies. There appears to be no influence of quantum interference.

Since the system in *cis* configuration is anisotropic with respect to the direction of the current flow, both flow directions were investigated. In Figure 7 the total currents in the *cis* configured 5-AGNR systems are depicted for the current flowing along the direction of the x component of the bond from O to C and opposite to that direction, so from left to right as shown in Figure 1. The corresponding plots for global and local currents with the reversed current can be found in the Supporting Information (Figures S10 and S11), where it can be seen that the flow is slightly more perturbed at lower biases.

**Figure 9:**
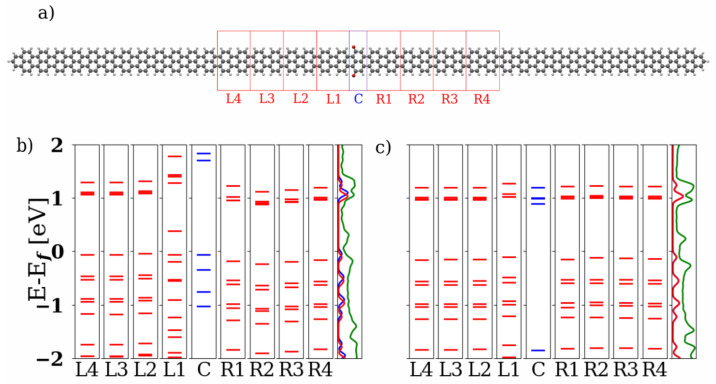
(**a**) Structure and partitioning of the *cis* 5-AGNR quinone system is depicted above. The carbon atoms are drawn in black, the hydrogen atoms in white and the oxygen atoms in red. The parts that were selected for the central scattering region (C) and the left (L1–L4) and right (R1–R4) cells are marked with red boxes in the image. Below: (**b**) The local energy spectrum of the quinone structure is shown and (**c**) the local energy spectrum of the hydroquinone structure. The local states are labeled with the corresponding sections. The rightmost sections show the PDOS for the leads in blue (left lead) and red (right lead) and the complete cluster in green.

The hydroquinone forms of the 5-AGNR systems show good level alignments in the local spectra for both *trans* and *cis* forms, as shown in Figure 8 and Figure 9. The energies of the lead units neighboring the central part of the scattering region are found to be strongly affected by the presence of the quinone group, for both the *trans* and *cis* forms. For the hydroquinone forms, the local spectra converge to their values within the lead already from the next neighbouring units, L2–L4 and R2–R4. In contrast, distortion in the local spectrum is observed up to L2 and R2 in the quinone-based devices.

Since the *cis* system is asymmetric along the transport direction, a much greater difference between the left and right sides can be seen in the quinone form (Figure 9b) than in the hydroquinone form (Figure 9c). The asymmetry is reflected in the local spectrum of the *cis* quinone device, where the levels in L1 are more disturbed than in R1. A slight asymmetry can even be observed in the PDOS of the leads. The levels of both hydroquinone forms align at energies somewhat above the Fermi level, which may explain the semi-conducting behaviour that is observed in the global transport (see Figure 7 above). Hybridization of the central region with the neighboring units does not lead to a major change in the levels from the central section. This may also contribute to a barrier for the transport. The clusters of states around the Fermi level as can be seen in the PDOS of all four AGNR systems seem to originate from the local states from the central region being gradually shifted as more units are included. For more details see Section S4 of the Supporting Information.

**Figure 10:**
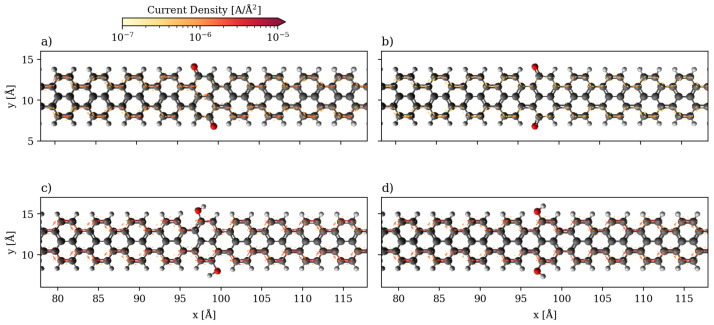
Quiver plot of the current densities at 1.0 V in the 5-AGNR systems projected onto real space on a grid at 1 $a_{0}$ spacing. The central scattering region are displayed with the same color-code as in Figure 1. (**a**) *trans* quinone system. (**b**) *cis* quinone system. (**c**) *trans* hydroquinone system. (**d**) *cis* hydroquinone system.

The local currents for the *trans* 5-AGNR systems shown in Figure 10 flow along the edges of the nanoribbon. Since after crossing the initial threshold for conductance the current flow does not qualitatively change for these systems, the local currents at a bias of 1.0 V are discussed here. The current density flows for the reverse direction can be found in the Supporting Information. The local currents for both directions appear to be qualitatively the same. In the quinone form there is a current visible travelling through the central bond between the two carbonyl functionalized rings. This current flows from one edge to cross the other. This may originate from the several possible resonance structures describing the central region of the *trans* quinone system, while only one exists to describe the *cis* quinone system. In order to avoid the perturbation from the carbonyl groups, this path may be preferable for the current. In the hydroquinone form, the perturbation is weaker and therefore the electrons do not have to deviate from the pathway along the edges.

In contrast to the local currents of the *trans* systems, those in the *cis* systems as seen in Figure 10 flow laminarly along the two edges. The flow appears to be weakest around the carbonyl groups. Since the quinone forms seem to lead to a greater perturbation of the current flow, it is possible to avoid these groups by flowing through the central rings crossing between the two edge currents. This increased flexibility in the current flow might explain the greater conductance in the *trans* 5-AGNR quinone system and the reduced current ratio from hydroquinone to quinone form in the *cis* form.

**Figure 11:**
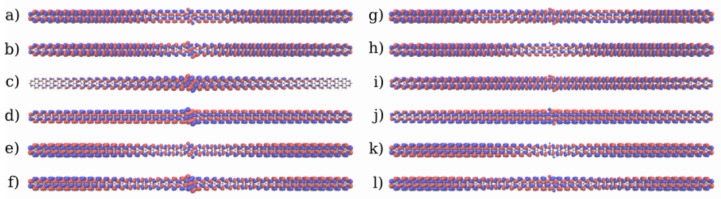
Frontier molecular orbitals of the *trans* 5-AGNR systems. (**a**–**f**): Orbitals of the quinone form from LUMO+2 to HOMO-2. (**g**–**l**): Orbitals for the hydroquinone form from LUMO+2 to HOMO-2. The phases of the orbitals are depicted in red and blue.

**Figure 12:**
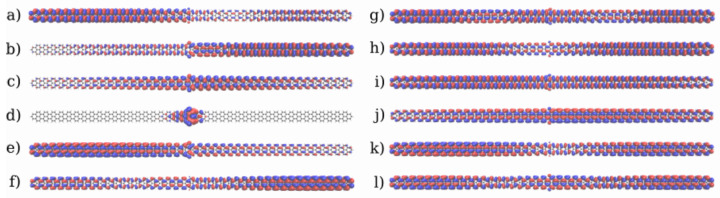
Frontier molecular orbitals of the *cis* 5-AGNR systems. (**a**–**f**): Orbitals of the quinone form from LUMO+2 to HOMO-2. (**g**–**l**): Orbitals for the hydroquinone form from LUMO+2 to HOMO-2. The phases of the orbitals are depicted in red and blue.

The molecular frontier orbitals of the 5-AGNR systems are shown in Figure 11 and Figure 12 respectively for the *trans* and *cis* systems. Those of the hydroquinone form are very strongly delocalized π orbitals. This may enable ballistic transport over an extended region around the scattering device. Yet, experimental work on related systems suggest this to be an unusually long distance, and the transport properties deep into the leads should not be overanalyzed [70,71,72]. On the other hand, the ballistic nature of transport in the leads would not affect the appearance of the mechanistic features inside the scattering region, as the leads serve as electron reservoirs. In the quinone form of the *trans* system the LUMO is partially localized in the center, which may impede the transport. As expected, in the quinone form of the *cis* system there are even more impediments to transport related to the frontier orbitals. Here, the HOMO is completely localised around a few rings in the center. Additionally, the HOMO-1 and LUMO+2 show a partial localization on the left and the HOMO-2 and LUMO+1 the same on the right.

## 4. Conclusions

In conclusion, we have investigated transport properties in pH-sensitive quinone-based devices with graphene nanoribbon leads. The investigations were performed through application of the NEGF formalism to calculate the global current through the Landauer equation and examination of the associated local current flow. For the 2-ZGNR system, the hydroquinone form is found to be more conductive in the bias window between 0.2–1.2 V. Beyond that a strong increase in conductance in the quinone system is observed. There are two currents flowing along each edge respectively without crossing and the perturbation of the quinone group plays a lesser role at higher biases. From these findings, the principle behind the change in conductance in the anthraquinone derivative observed by Darwish et al. [28] at different pH and previous theoretical work on the quantum interference in this system [30] could be replicated and expanded on.

The same design principle was proposed for similar devices based on the use of 5-AGNR leads. Theoretical investigations suggest that these reliably show strong current flow ratios between the hydroquinone and quinone form at all biases exceeding a conducting threshold. As in the ZGNR systems, the local currents are predicted to flow along the edges of the system. The *cis* configuration here is predicted to have a stronger on/off ratio. A possible explanation may be that in the *trans* configuration there is some transport possible between the two edges through the functionalized center, which enables the current to evade some of the perturbation from the carbonyl groups. 

## Data Availability

Data are contained within the article and Supplementary Materials.

## Supplementary Materials

The following supporting information can be downloaded at: https://www.mdpi.com/article/10.3390/nano13243085/s1, Figure S1: Convergence of buffer regions in ZGNR quinone system; Figure S2: Convergence of buffer regions in *trans* AGNR quinone system; Figure S3: Convergence of buffer regions in *cis* AGNR quinone system; Figure S4: Local energy spectra for ZGNR quinone system; Figure S5: Local energy spectra for ZGNR hydroquinone system; Figure S6: Local energy spectra for *trans* AGNR quinone system; Figure S7: Local energy spectra for *trans* AGNR hydroquinone system; Figure S8: Local energy spectra for *cis* AGNR quinone system; Figure S9: Local energy spectra for *cis* AGNR hydroquinone system; Figure S10: Reversed current densities in the *cis* AGNR quinone system; Figure S11: Reversed current densities in the *cis* AGNR hydroquinone system; Table S1: Occupation numbers for ZGNR quinone system; Table S2: Occupation numbers for ZGNR hydroquinone system; Table S3: Occupation numbers for *trans* AGNR quinone system; Table S4: Occupation numbers for *trans* AGNR hydroquinone system; Table S5: Occupation numbers for *cis* AGNR quinone system; Table S6: Occupation numbers for *cis* AGNR hydroquinone system.
