# Supplementary material for: Mechanistic Insights into Electronic Current Flow through Quinone Devices
Source: Nanomaterials (Basel). 2023 Dec 5;13(24):3085. doi: 10.3390/nano13243085 (PMC10745510; doi:10.3390/nano13243085)
Supplement: Supplementary file 1 [file nanomaterials-13-03085-s001.zip › nanomaterials-2730062-supplementary.pdf]

nanomaterials

Article

Mechanistic Insights into Electronic Current Flow through Quinone Devices

Lawrence Conrad [1,*], Isaac Alcón [2], Jean Christophe Tremblay [3] and Beate Paulus [1,*]

[1] Institut für Chemie und Biochemie, Freie Universität Berlin, 14195 Berlin, Germany
[2] Catalan Institute of Nanoscience and Nanotechnology (ICN2), Consejo Superior de Investigaciones Científicas (CSIC) and Barcelona Institute of Science and Technology (BIST), Campus Universitat Autònoma de Barcelona (UAB), Bellaterra,
08193 Barcelona, Spain; isaac.alcon@icn2.cat
[3] Laboratoire de Physique et Chimie Théoriques, Centre National de la Recherche Scientifique (CNRS)-Université de Lorraine, 1 Bd Arago, 57070 Metz, France; jean-christophe.tremblay@univ-lorraine.fr
* Correspondence: lawrence.conrad@fu-berlin.de (L.C.); b.paulus@fu-berlin.de (B.P.);
Tel.: +49-(0)30-838-53745 (L.C.); +49-(0)30-838-52051 (B.P.)

Keywords: non-equilibrium Green's function; Landauer formula; quinones; local currents; graphene nanoribbons; nanoelectronics

S1. Convergence of Buffer Zones

In the investigated clusters, a number of GNR unit cells need to be included between the leads and the actual region of interest in the center of the scattering region. The amount of these C_4H_2 buffer units required was calculated by increasing the amount used in the clusters and calculating the self-energies of the two units making up the leads. The energy differences are calculated and the average values, maximum and minimum values plotted. The same amount of buffer units are added between the respective leads and the corresponding end of the cluster.

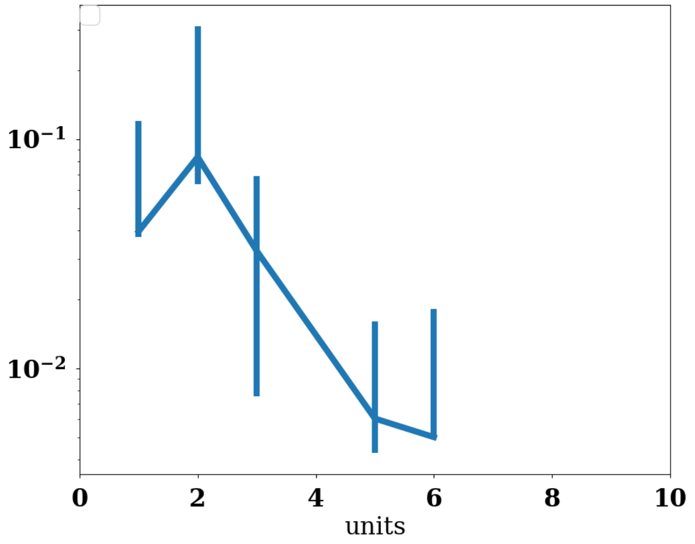

Figure S1. Average energy differences between self-energies of two halves of both leads of the ZGNR quinone with different amounts of C_4H_2 buffer units between the respective leads and both the center and the respective edges. The maximum and minimum values are included as boundaries for the error bars.

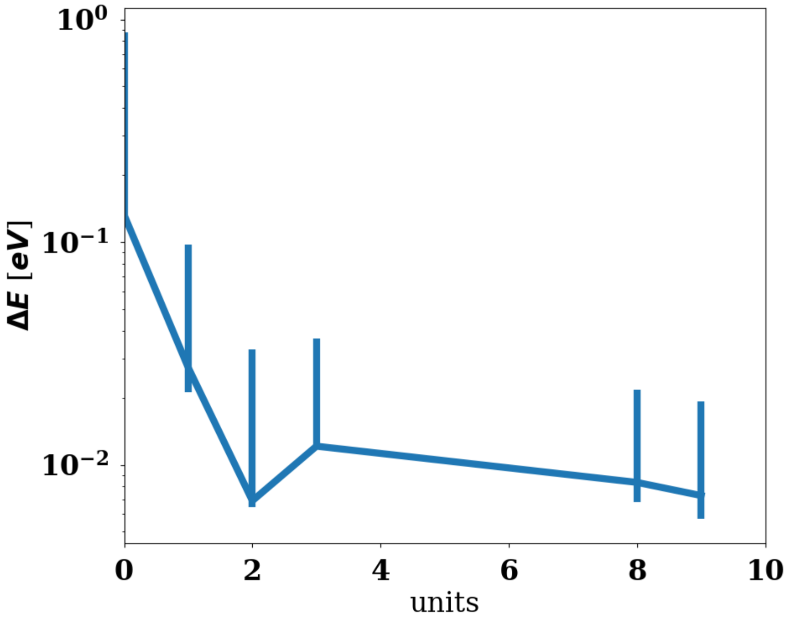

Figure S2. Average energy differences between self-energies of two halves of both leads of the *trans* AGNR quinone system with different amounts of C_4H_2 buffer units between the respective leads and both the center and the respective edges. The maximum and minimum values are included as boundaries for the error bars.

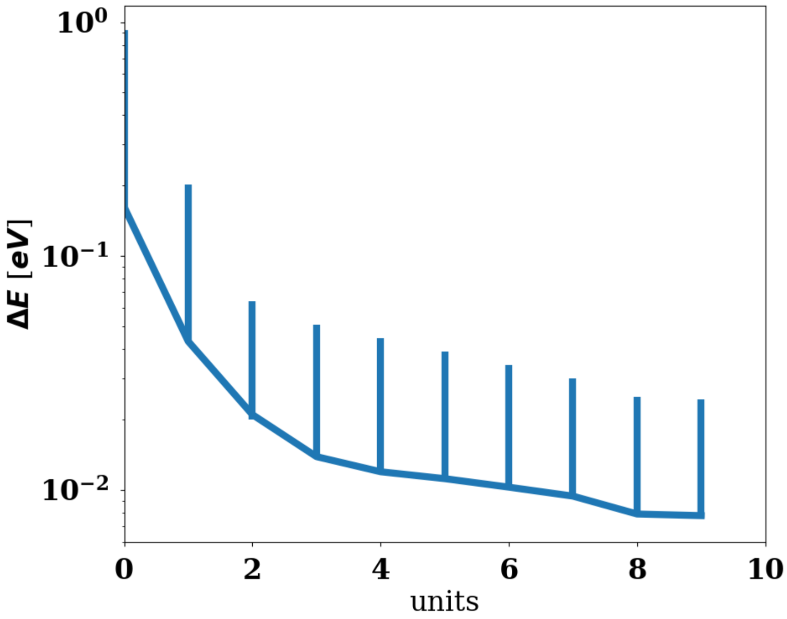

Figure S3. Average energy differences between self-energies of two halves of both leads of the *cis* AGNR quinone system with different amounts of C_4H_2 buffer units between the respective leads and both the center and the respective edges. The maximum and minimum values are included as boundaries for the error bars.

S2. Extraction of Hamilton Matrices from aomix Files

The aomix file from the Turbomole program package provides the molecular orbital coefficients C_{ij}, the corresponding eigen values E_j and the overlap matrix S. This allows the Hamilton matrix to be calculated from the secular equation in matrix form.

$$HC = SCE$$

$$H = SCEC^{-1} \tag{S1}$$

Since in the Turbomole program package, the atomic orbital basis is expressed in Cartesian form, but the molecular orbitals in spherical form, this causes the coefficient matrix C to be rectangular. However, despite extensive testing of numerous algebraic ideas, sensible results could not be obtained for the H matrix through use of a rectangular C matrix. The solution is to complete the space by adding a randomized molecular orbital vector to the matrix C after it has been orthonormalized with respect to the rest of the vectors (Gram-Schmidt process). Eigen values higher than the highest value resulting from the quantum chemical calculation are arbitrarily added by adding 0.5 eV to the current highest value. This process is repeated until the matrix C is square.

S3. Occupation Numbers

Table S1. Occupation numbers for the 2-ZGNR system in quinone form in D_{2h}.

D_{2h}	
a_g	92
b_{1g}	74
b_{2g}	15
b_{3g}	15
a_u	14
b_{1u}	16
b_{2u}	77
b_{3u}	89

Table S2. Occupation numbers for the 2-ZGNR system in hydroquinone form in C_{2h}.

C_{2h}	
a_g	166
b_g	31
a_u	30
b_u	166

Table S3. Occupation numbers for the *trans* 5-AGNR system in quinone form in C_{2h}.

C_{2h}	
a_g	566
b_g	104
a_u	105
b_u	565

Table S4. Occupation numbers for the *trans* 5-AGNR system in hydroquinone form in C_{2h}. The structure optimization results in a C_{2h} structure.

C_{2h}	
a_g	566
b_g	105
a_u	105
b_u	565

Table S5. Occupation numbers for the *cis* 5-AGNR system in quinone form in C_S. Due to the structure of the π electrons being ambiguous only a separation of σ and π electrons was used.

C_S	
a'	1131
a''	209

Table S6. Occupation numbers for the *cis* 5-AGNR system in hydroquinone form in C_{2v}. The structure optimization results in a C_{2v} structure.

C_{2v}	
a_1	629
a_2	84
b_1	502
b_2	126

S4. Evolution of Local States

In the AGNR systems the local energies from the central section are slightly shifted, when they interact with neighboring 5-AGNR units and it seems that the local energies from those units produce new levels that gradually converge to fit the complete PDOS of the systems. Meanwhile in the ZGNR systems, particularly the quinone form, the interaction with the first neighboring units already lead to strongly differing local energies. From this it can be concluded that there is strong coupling and hybridization occuring in these systems.

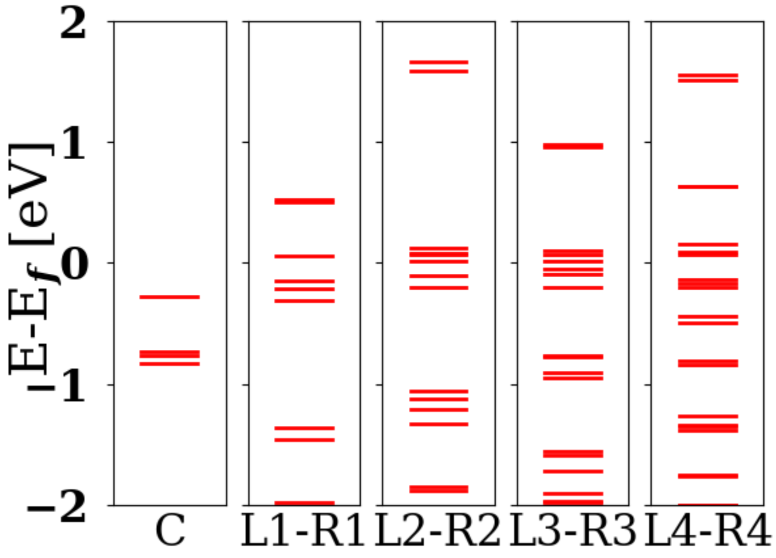

Figure S4. Local energy spectra for the ZGNR quinone system for the central region (C), the central region with the adjacent units L1 and R1 (L1-R1), all sections between and including L2 and R2 (L2-R2), all sections between and including L3 and R3 (L3-R3) and all sections between and including L4 and R4 (L4-R4).

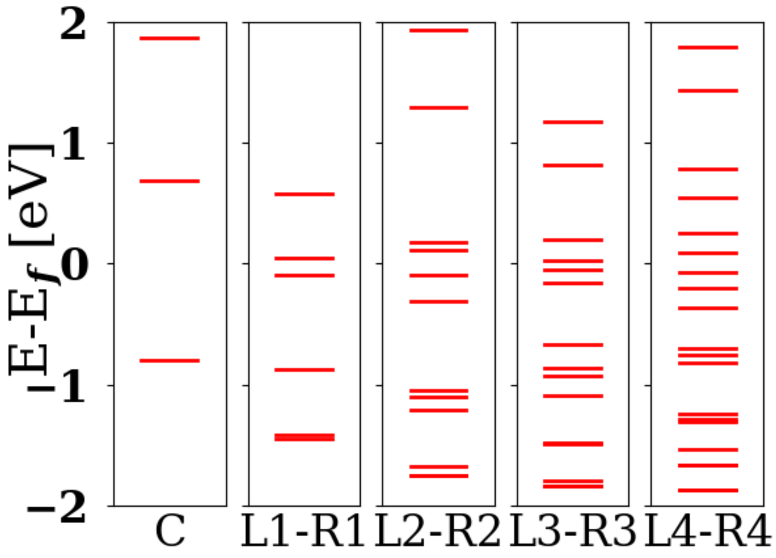

Figure S5. Local energy spectra for the ZGNR hydroquinone system for the central region (C), the central region with the adjacent units L1 and R1 (L1-R1), all sections between and including L2 and R2 (L2-R2), all sections between and including L3 and R3 (L3-R3) and all sections between and including L4 and R4 (L4-R4).

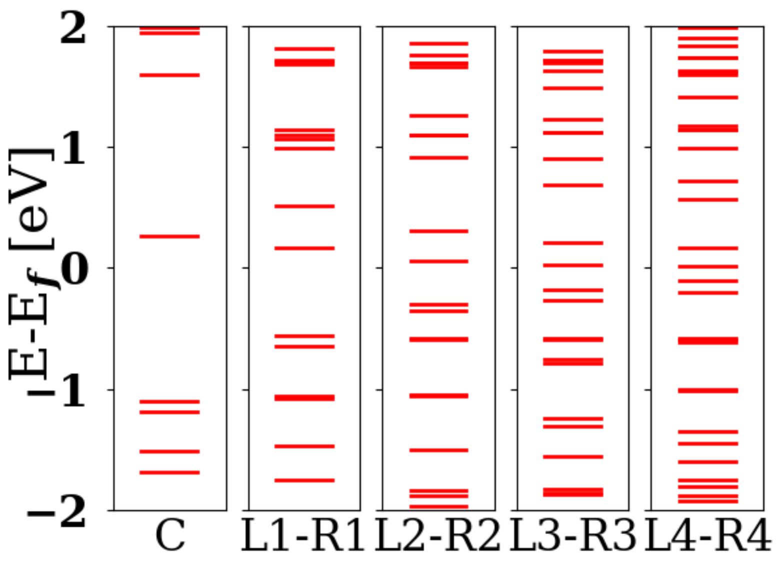

Figure S6. Local energy spectra for the *trans* AGNR quinone system for the central region (C), the central region with the adjacent units L1 and R1 (L1-R1), all sections between and including L2 and R2 (L2-R2), all sections between and including L3 and R3 (L3-R3) and all sections between and including L4 and R4 (L4-R4).

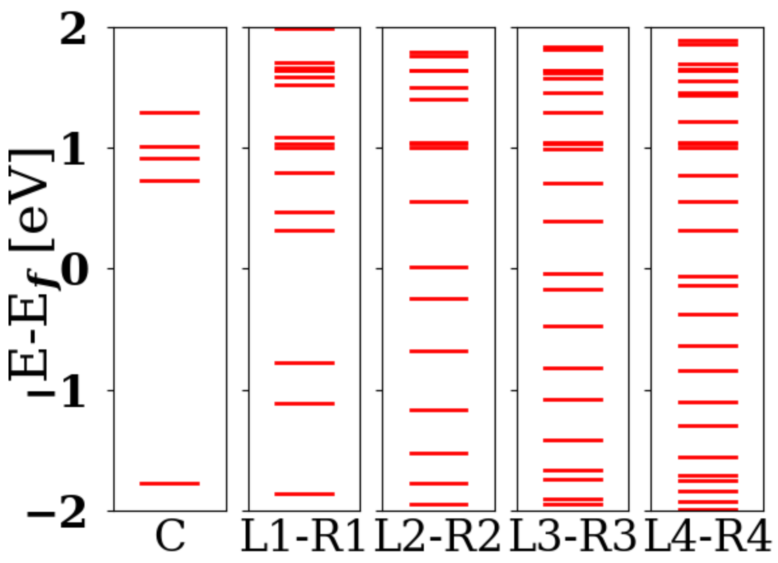

Figure S7. Local energy spectra for the *trans* AGNR hydroquinone system for the central region (C), the central region with the adjacent units L1 and R1 (L1-R1), all sections between and including L2 and R2 (L2-R2), all sections between and including L3 and R3 (L3-R3) and all sections between and including L4 and R4 (L4-R4).

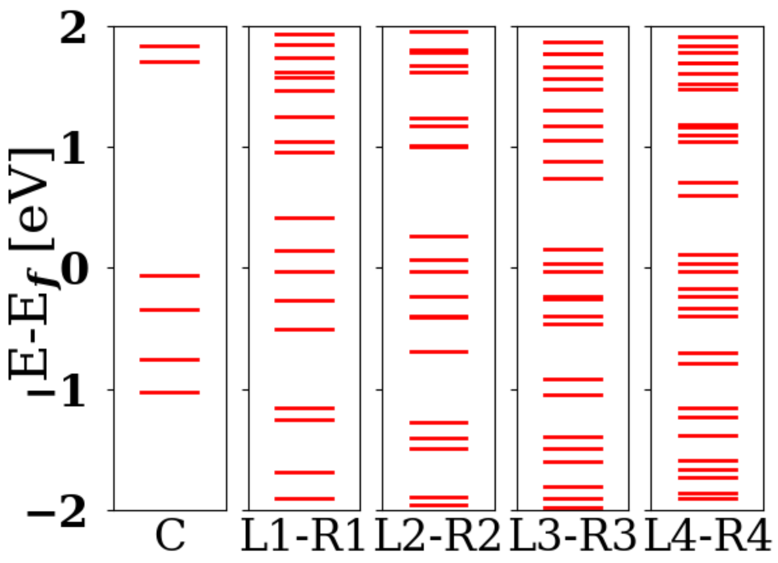

Figure S8. Local energy spectra for the *cis* AGNR quinone system for the central region (C), the central region with the adjacent units L1 and R1 (L1-R1), all sections between and including L2 and R2 (L2-R2), all sections between and including L3 and R3 (L3-R3) and all sections between and including L4 and R4 (L4-R4).

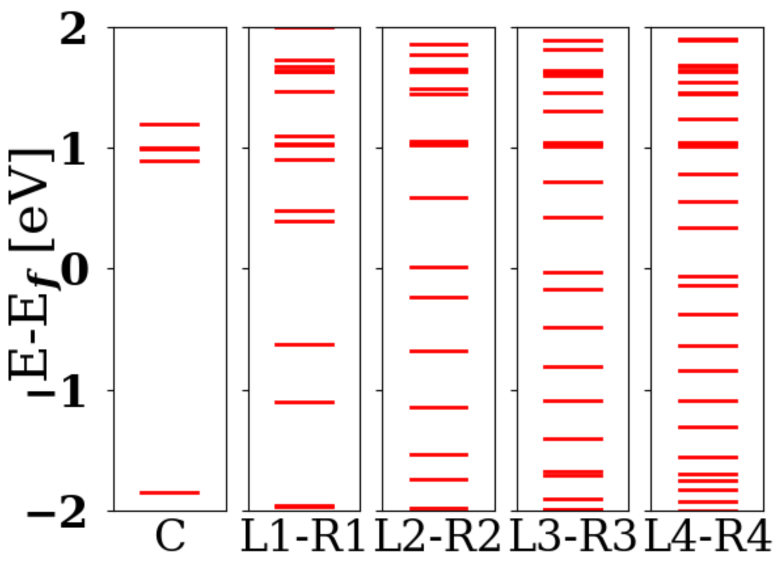

Figure S9. Local energy spectra for the *cis* AGNR hydroquinone system for the central region (C), the central region with the adjacent units L1 and R1 (L1-R1), all sections between and including L2 and R2 (L2-R2), all sections between and including L3 and R3 (L3-R3) and all sections between and including L4 and R4 (L4-R4).

S5. Reverse Currents in *cis* 5-AGNR Systems

Since the *cis* 5-AGNR systems are anisotropic in respect to the current direction, the reverse current was also investigated. It was found that qualitatively they do not differ from the current in the other direction.

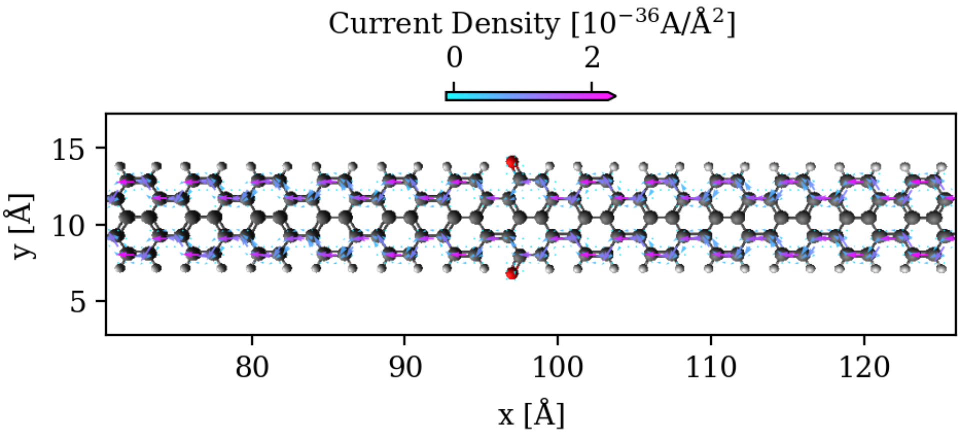

Figure S10. Quiver plot of the reversed current densities in the *cis* AGNR quinone system. They are projected onto real space on a cartesian grid at 1 a_0 spacing in all directions. Only the central scattering region is displayed. Carbon atoms are drawn in black, hydrogen atoms in white, and oxygen atoms in red.

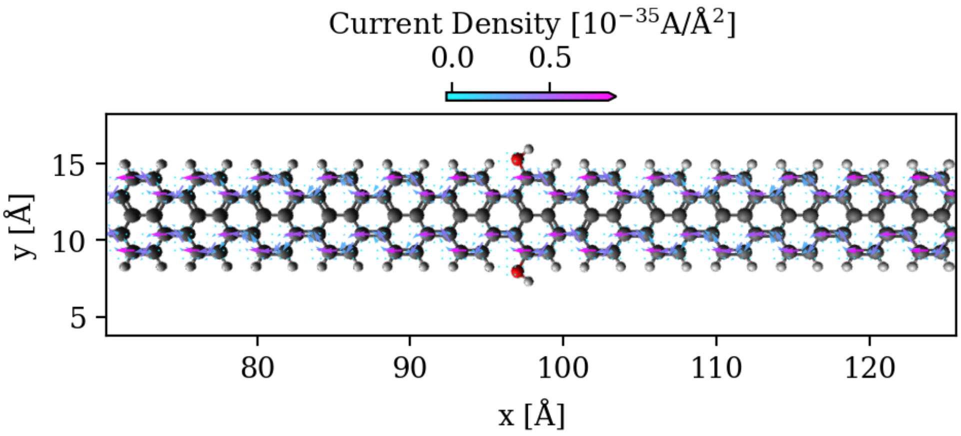

Figure S11. Quiver plot of the reversed current densities in the *cis* AGNR hydroquinone system. They are projected onto real space on a cartesian grid at 1 a_0 spacing in all directions. Only the central scattering region is displayed. Carbon atoms are drawn in black, hydrogen atoms in white, and oxygen atoms in red.
